# Genomic Determinants of De Novo Resistance to Immune Checkpoint Blockade in Mismatch Repair–Deficient Endometrial Cancer

**DOI:** 10.1200/PO.20.00009

**Published:** 2020-05-08

**Authors:** Doga C. Gulhan, Elizabeth Garcia, Elizabeth K. Lee, Neal I. Lindemann, Joyce F. Liu, Ursula A. Matulonis, Peter J. Park, Panagiotis A. Konstantinopoulos

**Affiliations:** ^1^ Harvard Medical School, Boston, MA; ^2^ Brigham and Women’s Hospital, Boston, MA; ^3^ Dana-Farber Cancer Institute, Boston, MA

## INTRODUCTION

Despite the success of programmed death 1 (PD-1)/PD ligand 1 (PD-L1) inhibitors in mismatch repair–deficient (MMRD) endometrial cancer (EC), many patients exhibit de novo resistance.^1,2^ To identify determinants of resistance to immune checkpoint blockade (ICB) in MMRD EC, we evaluated genomic data from patients who were enrolled in an investigator-initiated clinical trial of avelumab.^3^ In that study, avelumab met the prespecified criteria to be considered worthy of additional investigation in MMRD EC with an objective response rate of 26.7%. Responses to avelumab were observed regardless of PD-L1 expression, the presence or absence of tumor-infiltrating lymphocytes, multiple prior lines of therapy, and somatic or germline origin of MMRD, which suggests that baseline clinical and pathologic characteristics could not predict response. Here, we report Janus kinase 1 (*JAK1*) and β2-microglobulin (*B2M*) mutations and a higher number of insertions and deletions (indels) and exposure to an MMRD-associated mutational signature—Signature 20 in the Catalogue Of Somatic Mutations In Cancer—as candidate genomic determinants of de novo resistance to ICB in MMRD EC.

## PATIENTS AND METHODS

Formalin-fixed, paraffin-embedded samples were collected from patients enrolled in a phase II study of avelumab in mismatch repair–proficient (MMRP) and MMRD EC.^3^ Detailed information on sequencing and bioinformatic analyses is provided in the Data Supplement. The clinical trial was approved by the institutional review boards of all participating institutions and the US Food and Drug Administration (ClinicalTrials.gov identifier: NCT02912572). All procedures involving human participants were carried out in accordance with the Declaration of Helsinki. Written informed consent was obtained from patients before enrollment in the study as previously described.^3^

## RESULTS

### All Patients With MMRD EC Who Did Not Experience Response to Avelumab Harbored *JAK1* or *B2M* Mutations

Of 15 patients in the MMRD cohort—determined by immunohistochemistry (IHC)—who initiated avelumab therapy, targeted sequencing via OncoPanel was performed on 12 tumors (as a result of tissue availability). Ten of the 12 tumors were determined to be MMRD by OncoPanel on the basis of mutational signature analysis using two independent algorithms,^4,5^ which was consistent with the IHC determination. The remaining 2 tumors were determined to be MMRP by OncoPanel and microsatellite stable using polymerase chain reaction—that is, both OncoPanel and polymerase chain reaction were discordant with IHC—and none of them responded to avelumab. Of note, both tumors had a low number of indel mutations—only 4 indels in the first tumor and 2 indels in the second—compared with 34.5 indel mutations, on average, in the 10 tumors with concordant IHC and OncoPanel findings. In addition, both tumors harbored *TP53* mutations and extensive copy number alterations, rendering them most compatible with the copy number–high subgroup of endometrial carcinomas which are distinct from MMRD tumors. Therefore, these 2 tumors were more likely to be MMRP and were excluded from the analysis.

**FIG 1.**
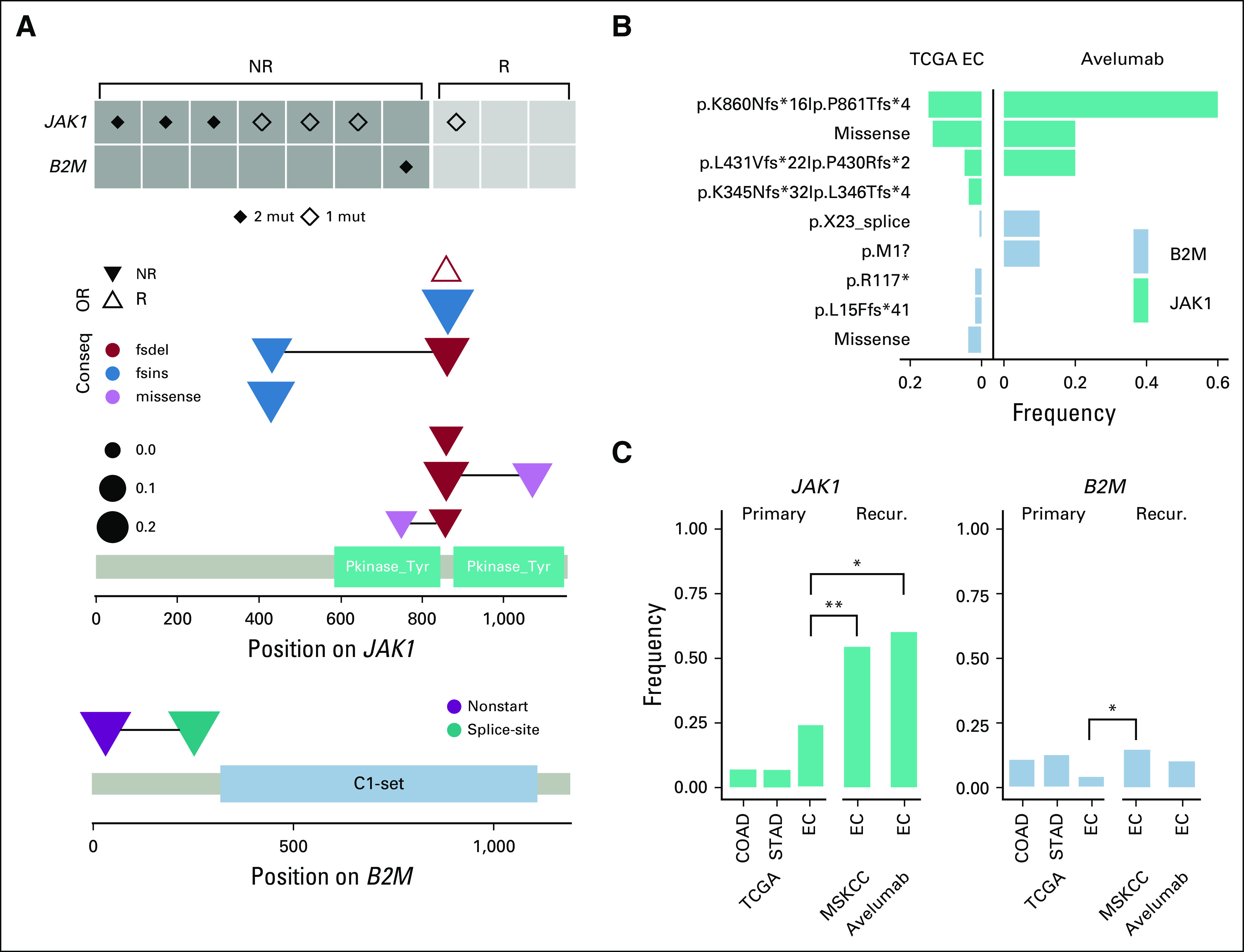
*JAK1* and *B2M* mutations. (A) Top: Mutation (mut) status for *JAK1* and *B2M* for all samples. The color of the tiles indicates response and the marker style indicates the number of mutations. Middle: Mutations on *JAK1* and *B2M* and their positions are shown for each patient. Patient’s response, the type, and allelic fraction of mutations are indicated by the marker style, color, and size, respectively. Bottom: Mutations on *B2M*. (B) Frequency of common *JAK1* and *B2M* mutations found in our data set are compared with the frequency in the mismatch repair–deficient (MMRD) endometrial cancers (ECs) from The Cancer Genome Atlas (TCGA). (C) Frequency of *JAK1* and *B2M* mutations are compared across tumor types (colorectal cancer [COAD], stomach adenocarcinoma [STAD], and EC) in the TCGA cohort, and the primary TCGA EC samples are compared with recurrent MSK-IMPACT and our data set. fs, frameshift; fsdel, frameshift deletion; fsins, frameshift insertion; NR, nonresponder; OR, objective response; R, responder; recur, recurrent. (*) *P* = .05-.01; (**) *P* = .01-.001.

Of the remaining 10 patients (Table 1) with tumors determined to be MMRD using both IHC and OncoPanel, 3 exhibited an objective response to avelumab (responders), whereas 7 did not (nonresponders). All 7 nonresponders harbored either *JAK1* (6 tumors) or *B2M* mutations (1 tumor), while only 1 of the 3 responders harbored a *JAK1* mutation (Fisher exact test, two-sided *P* = .067; Fig 1A). In addition, of the 7 nonresponders, 4 harbored two mutations of *JAK1* (3 tumors) or *B2M* (1 tumor), possibly reflecting biallelic inactivation of these genes. Conversely, none of the 3 responders exhibited two mutations in either gene (Fisher exact test, two-sided *P* = .2).

**TABLE 1.**
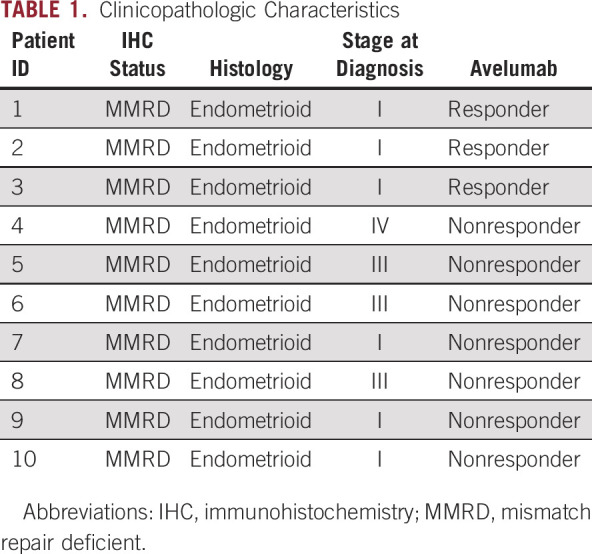
Clinicopathologic Characteristics

Type of mutation, allelic fraction, and position on *JAK1* and*B2M* genes is shown in Figure 1A. All *JAK1* mutations were frameshift (deletions or insertions), with the exception of two missense mutations that occurred together with frameshift mutations: a missense mutation Q750R on the pseudokinase domain (exon 16) and a missense mutation L1071P toward the end of the kinase domain (exon 23). Frameshift *JAK1* mutations involved the hotspot position K860/P861 (deletions in 5 tumors and insertion in 1 tumor) and the hotspot position P430/L431 (insertions in 2 tumors). The sole tumor with mutation in *B2M* was a nonresponder that harbored two *B2M* mutations previously reported in The Cancer Genome Atlas (TCGA): a *B2M* c.68-2A>G splice-site mutation (19 of 10,953 patients in TCGA across all tumor types) and a p.M1? mutation changing the start codon.

### Frequency of *JAK1* and *B2M* Mutations Was Higher Compared With TCGA Data

As shown in Figure 1B, the frequency of *JAK1* and *B2M* mutations in our data set was higher compared with the frequency of these mutations among the MMRD ECs included in TCGA. For example, in our trial, 60% (6 of 10 MMRD patients) harbored *JAK1* frameshift mutations involving the hotspot K860/P861 position compared with only 14.8% of patients with MMRD cancers in the TCGA EC data set (Fig 1B). Overall, 70% of patients (7 of 10) exhibited frameshift *JAK1* mutations in our trial compared with only 23.8% of patients with MMRD cancers in the TCGA EC data set (Fig 1C).

This difference may reflect the fact that patients with MMRD tumors included in the TCGA EC data set were all newly diagnosed EC cases, regardless of whether their tumors eventually recurred. On the contrary, our data set—that is, patients enrolled in the avelumab study for recurrent endometrial cancer—consisted solely of patients whose tumors recurred. Furthermore, in another data set (MSK-IMP), which included 29 patients with recurrent MMRD ECs (defined by IHC),^6^ the overall incidence of frameshift *JAK1* mutations was 51.7%—significantly higher than that in the TCGA data set, but comparable with that in our data set (Fig 1C). A similar trend was also observed with *B2M* mutations (Fig 1C).

### Number of Total Indel Mutations and Exposure to Mutational Signatures of MMRD

**FIG 2.**
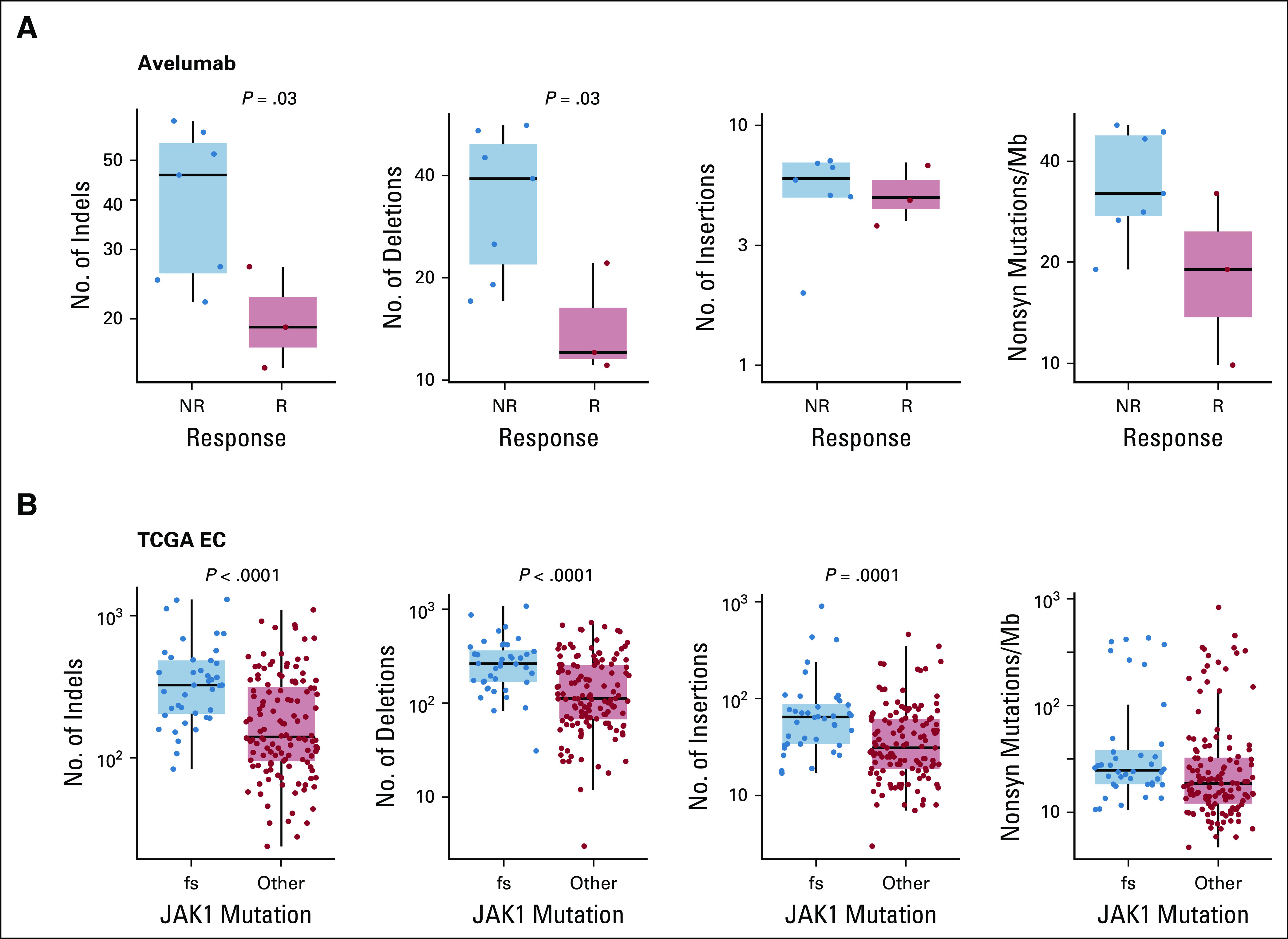
Comparison of indel and mutation counts. (A) Number of indels, deletions, insertions, and tumor mutational burden, defined as the number of nonsynonymous mutations per Mb for responders (R) and nonresponders (NR) in our data set. (B) Same as panel A comparing patients with endometrial cancer (EC) in The Cancer Genome Atlas (TCGA) data set with and without frameshift *JAK1* mutations. *P* values are calculated with double-sided *t* test and are shown in panels whenever they are < .05. fs, frameshift.

As shown in Figure 2A, nonresponders had a significantly higher number of total indels compared with responders (two-sided *t* test; *P* = .03; bootstrapping *P* = .05). Nonresponders had a significantly higher number of total deletion mutations compared with responders (*P* = .03), but the number of total insertion mutations was not different (Fig 2A). There was no difference in the total number of nonsynonymous mutations, also defined as tumor mutational burden (TMB), between nonresponders and responders (Fig 2A). To assess whether a higher number of total indels correlated with the presence of *JAK1* mutations in MMRD EC, we evaluated this association in the TCGA data set. Indeed, the presence of *JAK1* mutations in the MMRD tumors of the TCGA EC data set was associated with a significantly higher number of total indel mutations, number of deletions, and number of insertions, but not higher TMB (Fig 2B), which suggests that a higher number of indels, but not TMB, may be tracking the presence of *JAK1* mutations in MMRD EC.

**FIG 3.**
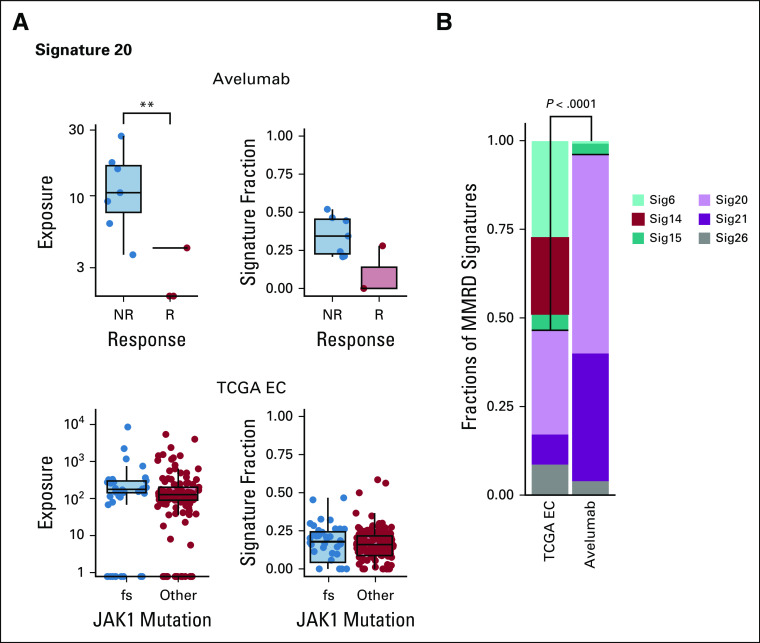
Comparison of mutational signatures. (A) Exposure (left) and fraction (right), defined as the exposure divided by the total number of single-nucleotide variants of signatures 20. Top: Blue and red boxes and points indicate nonresponders (NR) and responders (R), respectively, in our data set. Bottom: Blue and red boxes and points indicate cases with and without frameshift (fs) *JAK1* mutations in whole-exome sequencing data from The Cancer Genome Atlas (TCGA). (**) *P* = .01-.001. (B) The average fractions of mismatch repair–deficient (MMRD) signatures in avelumab and the TCGA data set are compared. To calculate the fraction, the exposure of each MMRD signature is divided by the sum of exposures of all MMRD signatures (the contribution of the non-MMRD signatures is not shown as they did not vary significantly across data sets). EC, endometrial cancer.

In addition, we assessed whether the presence of mutational signatures of MMRD,^7-10^ namely Signatures 6, 14, 15, 20, 21, and 26 in the Sanger COSMIC catalog, was associated with response of MMRD ECs to avelumab. As shown in Figure 3A and the Data Supplement, only Mutational Signature 20 was enriched in nonresponders compared with responders (two-sided *t* test *P* = .009; bootstrapping *P* = .014). The fraction of Signature 20 (Fig 3B) was also higher in nonresponders (fold change in mean value, 3.7; two-sided *t* test *P* = .08; bootstrapping *P* = .09). Unlike total indel count, Mutational Signature 20 did not correlate with the presence of *JAK1* mutations in MMRD ECs in the TCGA data set (Fig 3A), which suggests that the presence of Mutational Signature 20 may be tracking alternative mechanisms of resistance. Finally, as shown in Figure 3B, the mutational signature composition of the samples in our trial was different from that of the TCGA, with a higher fraction of T>C mutations (Signatures 20, 21, and 26) being enriched (*P* < .0001) in our trial.

## DISCUSSION

Mutations in genes that are involved in the interferon signaling and antigen-presentation pathways are well-characterized mechanisms of resistance to ICB.^11-13^ Abrogation of interferon-gamma signaling via loss-of-function mutations in *JAK1* and *JAK2* has been previously shown to allow escape from interferon-induced inhibition of growth and thus confer resistance to PD-1/PD-L1 and cytotoxic T-lymphocyte–associated protein 4 blockade.^11,12^ Dysregulation of antigen-processing machinery via mutations in *B2M*—a gene involved in proper major histocompatibility complex class I folding and transport to the cell surface that is required for CD8 T-cell recognition—is another well-recognized mechanism of resistance to ICB.^13^ To our knowledge, this is the first study to report *JAK1* and *B2M* mutations in association with response to ICB in MMRD EC and the first to report these as a mechanism of de novo—as opposed to acquired—resistance.

It is important to underscore that, outside of the context of response to ICB, frameshift *JAK1* mutations have been previously reported to occur de novo in EC and have been functionally characterized to abrogate interferon-γ signaling as well as contribute to tumor immune evasion in this disease.^14-16^ In this regard, it was not surprising to find that the incidence of *JAK1* mutations in our data set, which included only patients whose tumors eventually recurred, was significantly higher than that in the TCGA EC data set, which included all comers with newly diagnosed EC, suggesting that these mutations may be enriched in MMRD ECs which eventually recur.

Finally, exploratory analysis demonstrated a significantly higher number of total indels in avelumab nonresponders. It is well established that indel mutations contribute to the generation of neoantigens, which increase tumor immunogenicity and the likelihood of response to ICB.^17^ However, more indels also increase the likelihood that important genes, such as *JAK1* and *B2M*, that are necessary for effective antitumor immune response may become truncated and thereby contribute to resistance to ICB. Taken together, whereas the presence of a higher number of indels in MMRD tumors compared with MMRP tumors explains their higher immunogenicity and response to ICB, a higher number of indels among MMRD tumors may drive the presence of *JAK1* mutations and resistance to ICB.
